# Effects of group mindfulness-based cognitive therapy and group cognitive behavioural therapy on symptomatic generalized anxiety disorder: a randomized controlled noninferiority trial

**DOI:** 10.1186/s12888-022-04127-3

**Published:** 2022-07-19

**Authors:** Si-si Jiang, Xue-hua Liu, Nan Han, Hai-jing Zhang, Wu-xiang Xie, Zhi-juan Xie, Xin-yuan Lu, Xuan-zi Zhou, Yu-qi Zhao, Ai-deng Duan, Shu-qin Zhao, Zhi-cheng Zhang, Xue-bing Huang

**Affiliations:** 1 NHC Key Laboratory of Mental Health (Peking University), Peking University Sixth Hospital, Peking University Institute of Mental Health, National Clinical Research Center for Mental Disorders (Peking University Sixth Hospital), Beijing, China; 2 Beijing Chang Ping Hospital of Integrated Chinese and Western Medicine, Beijing, China; 3 Peking University Clinical Research Institute, Peking University Health Science Center, Beijing, China; 4 Peking University People’s Hospital, Beijing, China

**Keywords:** Mindfulness, Generalized anxiety disorder, Cognitive behavioural therapy, Treatment

## Abstract

**Background:**

Mindfulness-based cognitive therapy (MBCT) is a promising alternative treatment for generalized anxiety disorder (GAD). The objective of this study was to examine whether the efficacy of group MBCT adapted for treating GAD (MBCT-A) was noninferior to group cognitive behavioural therapy (CBT) designed to treat GAD (CBT-A), which was considered one of first-line treatments for GAD patients. We also explored the efficacy of MBCT-A in symptomatic GAD patients compared with CBT-A for a variety of outcomes of anxiety symptoms, as well as depressive symptoms, overall illness severity, quality of life and mindfulness.

**Methods:**

This was a randomized, controlled, noninferiority trial with two arms involving symptomatic GAD patients. Adult patients with GAD (*n* = 138) were randomized to MBCT-A or CBT-A in addition to treatment as usual (TAU). The primary outcome was the anxiety response rate assessed at 8 weeks after treatment as measured using the Hamilton Anxiety Scale (HAMA). Secondary outcomes included anxiety remission rates, scores on the HAMA, the state-trait anxiety inventory (STAI), the Hamilton Depression Scale (HAMD), the Severity Subscale of the Clinical Global Impression Scale (CGI-S), and the 12-item Short-Form Health Survey (SF-12), as well as mindfulness, which was measured by the Five Facet Mindfulness Questionnaire (FFMQ). Assessments were performed at baseline, 8 weeks after treatment, and 3 months after treatment. Both intention-to-treat (ITT) and per-protocol (PP) analyses were performed for primary analyses. The χ2 test and separate two-way mixed ANOVAs were used for the secondary analyses.

**Results:**

ITT and PP analyses showed noninferiority of MBCT-A compared with CBT-A for response rate [ITT rate difference = 7.25% (95% CI: -8.16, 22.65); PP rate difference = 5.85% (95% CI: − 7.83, 19.53)]. The anxiety remission rate, overall illness severity and mindfulness were significantly different between the two groups at 8 weeks. There were no significant differences between the two groups at the 3-month follow-up. No severe adverse events were identified.

**Conclusions:**

Our data indicate that MBCT-A was noninferior to CBT-A in reducing anxiety symptoms in GAD patients. Both interventions appeared to be effective for long-term benefits.

**Trial registration:**

Registered at chictr.org.cn (registration number: ChiCTR1800019150, registration date: 27/10/2018).

**Supplementary Information:**

The online version contains supplementary material available at 10.1186/s12888-022-04127-3.

## Background

Generalized anxiety disorder (GAD) is one of the most prevalent psychiatric disorders and is characterized by chronic and persistent worry [1, 2]. It is associated with substantial costs for the individual as well as families and society [3]. However, GAD is the least successfully treated anxiety disorder [4], and approximately 50% of patients do not respond to first-line treatments (e.g., pharmacotherapy or cognitive behavioural therapy) [1, 5]. Only 30–50% of GAD patients experience remission [6, 7], and many remitted patients experience residual symptoms over time [3].

Due to the disadvantages of pharmacotherapy, including side effects, premature discontinuation, and a significant risk of relapse [8, 9], a large proportion of patients prefer psychotherapy to pharmacotherapy. Among the various forms of psychotherapy, cognitive behavioural therapy (CBT) is considered a first-line treatment for GAD [10, 11]. CBT should be administered by therapists with a background in psychiatry or psychology. In addition to a shortage of trained CBT therapists, individual sessions are expensive in health care systems with limited resources [12]. Consequently, CBT is not widely offered in clinical practice. Thus, more evaluation of other potential alternative treatments for GAD is needed.

Mindfulness is the awareness that emerges through intentionally focusing, in a nonjudgmental way, on how things are in the present moment [13]. Mindfulness-based cognitive therapy (MBCT), which combines the practice and principles of mindfulness with CBT components, was originally developed to prevent the recurrence of depression for patients in recovery [14]. MBCT is currently recommended by the NICE guidelines for the treatment of recurrent depression [13–17]. In recent years, several studies have shown that, except for relapse prevention strategies, mindfulness-based intervention (MBI), including MBCT, may also reduce acute symptoms of anxiety and depression. A meta-analysis of 39 studies with a total of 1140 participants demonstrated that MBI had significant effects on the reduction of anxiety symptoms in persons with anxiety disorders [18]. This meta-analysis highlights that future studies should make direct comparisons between the effectiveness of conventional CBT and MBI [18].

Although some randomized controlled trials (RCTs) [19] have evaluated the effects of MBI for treating GAD in adults, most of these had small sample sizes [20, 21], included participants with heterogeneous diagnoses (including anxiety disorders other than GAD) [22–24], or assessed MBIs without an active control [25]. Three well-conducted early RCTs compared the efficacy of MBI and CBT in patients with GAD [26–28]. However, these studies all included only psychoeducation groups using CBT principles, which are assumed to be low-intensity interventions, as active controls to suppress the nonspecific effects of the treatment. Conventional group CBT is assumed to be a high-intensity intervention and a first-line treatment for GAD patients [27]. CBT is considered to be the gold standard for evaluating the efficacy of new and promising interventions [29]. To the best of our knowledge, evidence directly comparing the efficacy of MBI and conventional group CBT for the treatment of GAD is lacking.

Our primary objective was to compare MBCT-A with gold standard psychological treatment CBT-A to test the hypothesis that the anxiety response rate achieved with MBCT-A would not be more than 10% worse than that achieved with CBT-A. We hypothesized that MBCT-A would be noninferior to CBT-A in improving symptoms of anxiety. Our other objectives were to explore the effects of MBCT-A and CBT-A in terms of psychic and somatic anxiety symptoms, state and trait anxiety symptoms, depression symptoms, overall illness severity, quality of life and mindfulness.

## Methods

### Trial design and participants

This study is a randomized, controlled, noninferiority trial with two groups: MBCT-A vs. CBT-A. Participants were recruited from the Outpatient Department of the Sixth Hospital of Peking University from November 2018 to November 2019 via (a) posters distributed in outpatient clinics and (b) recommendations from psychiatrists who worked in the Sixth Hospital of Peking University but were not involved in the study. All patients concurrently continued their regular outpatient psychiatry visits for medication management during the study period (treatment-as-usual, TAU) at the Sixth Hospital of Peking University. The regular outpatient psychiatry visits did not include psychotherapy, and the average consulting time was approximately 10 min per patient, with an average of one visit every two weeks.

All interested participants were assessed for eligibility. First, a trained research assistant screened all interested participants by telephone or in-person appointments using HAMA and a brief structured questionnaire. Additionally, the research assistant discussed the process of the study with interested participants. Next, for patients who passed the first screening, diagnostic screenings were independently performed by an attending psychiatrist in accordance with the Diagnostic and Statistical Manual of Mental Disorders, fourth edition (DSM-IV). Finally, the principal investigator (B.X.H.) conducted a final screening using the study inclusion and exclusion criteria to determine eligibility. Informed consent forms were obtained from all eligible participants.

We used the following inclusion criteria: (a) aged 18–65 years; (b) diagnosis of GAD based on DSM IV; (c) score ≥ 14 on the Hamilton Anxiety Rating Scale (HAMA) [30]; (d) medication at a stable dose for ≥ 1 month; and (e) ability to understand and communicate in Chinese. The exclusion criteria were as follows: (a) diagnosis of any past or present organic mental disorder, schizophrenia, schizoaffective disorder, major depression disorder, bipolar disorder or any other type of anxiety disorder according to the DSM-IV; (b) abuse of alcohol or other substances in the past 12 months; (c) any conditions that were potentially life-threatening or could severely limit participation (e.g., serious suicidal ideation, antisocial personality disorder, severe or unstable medical illness, pregnancy, breastfeeding); (d) current engagement in psychological treatment for GAD; and (e) a history of attending 4 or more mindfulness sessions in the past 2 years. Participants were withdrawn from the study if they (a) had any suicidal behaviour or suicide attempts; (b) withdrew their informed consent; or (c) were absent for more than three therapy sessions during the study period. The only difference in inclusion and exclusion criteria between this trial and the previous group CBT trial for GAD [31] was those patients with a history of attending 4 or more mindfulness sessions in the past 2 years could be excluded from this trial.

### Interventions

#### Intervention: MBCT-A

The MBCT-A protocol largely followed the classical MBCT manual described by Segal, Williams, and Teasdale [14, 32]. The classical MBCT intervention comprises individual precourse orientation, 8 weekly group classes and one “retreat in silence” day. In addition, participants are asked to engage in daily homework guided by audio recordings after weekly sessions.

In the MBCT-A manual, we made several adaptations to render the MBCT appropriate for treating GAD. First, the cognitive components of MBCT dealing with depression were replaced by components dealing with anxiety, which included psychoeducation about anxiety and automatic anxiety thoughts in session four, identifying warning signs for anxiety in session six. Second, the content of the precourse orientation was integrated into session one, a one-day retreat was shortened to 4 h and integrated into session six, and daily audio homework exercises were shortened to 30 min a day. Third, loving-kindness meditation was introduced in session two to cultivate kindness within GAD patients themselves and compassion for the somatic and psychic anxiety symptoms of GAD [33].Practices in the MBCT-A included mindful eating, body scans, sitting meditation, loving-kindness meditation, 3-min breathing space exercises, mindful yoga and mindful walking. These changes were developed on the basis of (1) the dissimilar characteristics between GAD and depression, (2) the different needs when attempting to improve current anxiety symptoms vs. preventing a relapse of depression, and 3) a literature review [26, 27, 33] and clinical experience. The first author (S.S.J) and co-first author (X.H.L) wrote the adapted manualized protocol. The session summary can be found in the Online Supplementary Protocol.

Qualified instructors with more than two years of experience delivered the MBCT-A program and were supervised by Dr. S. Helen Ma and Dr. Yen-Hui Lee, who both were Oxford mindfulness centres and approved the MBCT Teacher Trainer & Supervisor. The MBCT-A intervention consisted of weekly sessions for 8 weeks, each lasting 2 h involving up to 20–25 participants. All participants were instructed to report their daily mindfulness practice via a messaging and social media application (WeChat).

### Control: CBT-A

The CBT-A group, as the active control intervention with the same protocol as in a previous trial [31], has been validated as effective in patients with GAD and is considered a high-intensity psychotherapy intervention for GAD. The CBT-A program followed a manualized protocol originally authored by the corresponding author (X.B.H.) and that had been used successfully in previous clinical trials [31]. The main aim of CBT is to change or challenge the “dysfunctional” thoughts related to generalized anxiety and to introduce the participant to various relaxation techniques.

Two qualified therapists with either a psychiatry or psychotherapy background led each CBT-A group. The corresponding author X. B. H supervised the CBT-A therapists during the study period. Participants in the CBT-A group attended weekly 1.5 h sessions over 8 weeks, with 10–15 participants in each group. Each CBT-A session had a particular theme. Weekly homework was assigned at the end of each session and was handed in to the therapists and discussed in the following session. The session summary can be found in the Online Supplementary Protocol.

### Outcomes

The outcome measures were collected at baseline (T1), week 8 (postintervention, T2), and at a 3-month follow-up assessment (T3). Four trained psychiatric residents who were blinded to the patient’s treatment allocation conducted patient assessments. Demographic and baseline clinical information, including age, sex, education history, marital status, ethnicity, residential location, religious beliefs, age of onset, course of GAD, and use of medication, was collected using a questionnaire.

The primary outcome was response rate: percentage of participants who received a ≥ 50% HAMA decrease relative to the baseline at 8 weeks. The secondary outcomes included the response rate at the 3-month follow-up, remission rate at the 8-week and 3-month follow-ups, and the symptoms of total, psychological, and somatic anxiety, state and trait anxiety, depression, overall illness severity, quality of life and mindfulness. Any severe adverse events were observed throughout the whole intervention process.

### Instruments

The HAMA [30] is a 14-item scale used to assess symptom severity in patients with anxiety disorders and includes two subscales: the psychic anxiety subscale and the somatic anxiety subscale. In the present study, anxiety response was defined as a ≥ 50% decrease relative to the baseline, and anxiety remission was defined as a HAMA total score of less than 7 [31, 34]. Total, psychological, and somatic anxiety symptoms were measured by HAMA and subscales. In this study, depressive symptoms were measured by the 17-item Hamilton Depression Scale [35], overall illness severity was measured by the Severity Subscale of the Clinical Global Impression Scale (CGI-S) [36], and quality of life was measured by the 12-item Short-Form Health Survey (SF-12) [37]. Trait mindfulness was measured using the 39-item Five Facet Mindfulness Questionnaire (FFMQ) [38], on which a higher total score (range 39–195) suggests a higher level of mindfulness. This scale has been translated into Chinese and validated.

### Sample size

We calculated the sample size to investigate whether MBCT-A was not inferior to CBT-A. Because no previous studies directly compared MBCT with high-intensity CBT for the treatment of GAD, we used the findings from two studies – one that compared the effects of CBT and medication [31] and another that compared the effects of MBCT and medication [39] – to assume the response rate of our sample size calculation. According to these two previous studies, we assumed that the response rate for MBCT was 76.7% [31], the response rate for CBT was 63.6% [29] and the noninferiority margin was 10.0%. With a type I error-set of 2.5% (one-sided) and a type II error-set of 20%, we calculated the sample size per group to be 61 participants. With a presumed drop-out rate of 13%, we aimed to recruit 69 participants per group. For this sample size calculation, PASS 15.0 (NCSS, Kaysville, UT, USA) for noninferiority trials was used.

### Randomization—sequence generation, allocation concealment, implementation

Randomization was performed using computer-generated random numbers that were generated by an independent statistician. A block randomization procedure (blocks of 6) with no stratification was used. Those involved in the trial were blinded to the block sizes.

**Fig. 1:**
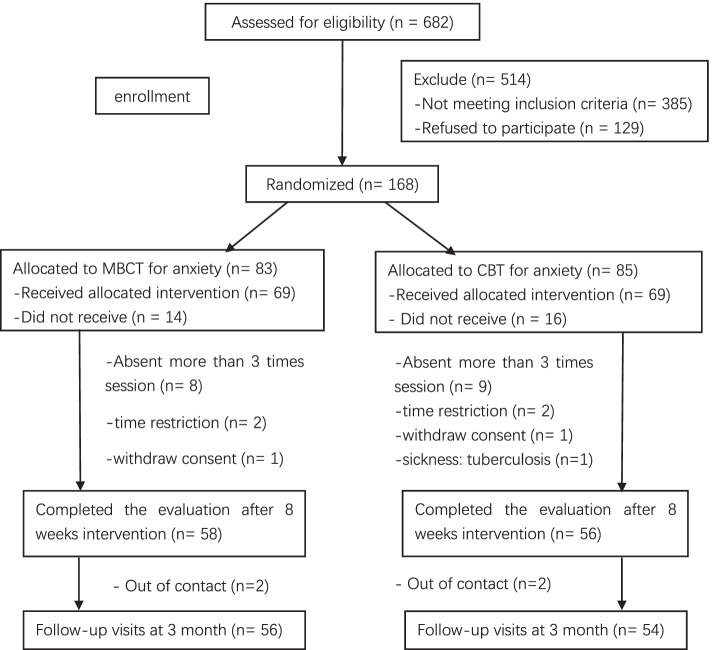
Participant flow chart

Sealed envelopes were used to conceal the randomization sequence. The intervention types were written on sheets of paper that were placed inside opaque envelopes. After the informed consent forms were signed, a trained research assistant opened the envelopes in order and noted the group assignment for the corresponding participants. The participants were informed of their group allocation immediately after randomization, and they were notified that the treatments received in both study groups could be helpful in improving anxiety symptoms. The participants who agreed to receive the allocation were informed to wait for notice of the group starting from the research assistant. The study flow diagram is shown in Fig. 1.

### Blinding

The researchers were blinded to the randomization. The independent assessors were blinded to the treatment allocation. The analyst was blinded to the identity of the participants until the results were finalized.

### Statistical methods

Statistics were conducted using IBM SPSS Statistics ver. 22. The baseline characteristics of the two groups were compared using an independent samples t-test or Mann–Whitney’s U test for continuous variables and the χ2 test for categorical variables. For the primary outcome, both intention-to-treat (ITT) (including the participants who attended at least one session) and per-protocol (PP) analyses (including the participants who attended at least five out of eight sessions) were performed, and the 95% CI level was used to interpret the differences in MBCT-A and CBT-A response rates at 8 weeks. For the secondary analyses, only PP analyses were performed. We used the χ2 test to analyse the response rates at the 3-month follow-up and remission rates in the two groups at each assessment time (8 weeks after the start of the treatment, 3-month follow-up). The effect size estimates were presented using Cohen’s d and were interpreted as small effects (0.2–0.5), moderate effects (0.5 to 0.8), and large effects (≥ 0.8) [40]. We performed separate two-way mixed ANOVAs to compare the mean differences in all other secondary outcomes. Group (MBCT-A vs. CBT-A) was used as a between-subjects factor, and time (baseline, 8 weeks after treatment onset, 3-months follow-up) was used as a within-subjects factor. The Bonferroni post hoc test was used for post hoc comparisons at each assessment. Partial eta squared (η2p) values were calculated for all significant findings. The significance level was set at *p* < 0.05.

## Results

### Baseline characteristics and dropout rates

Out of the 682 screened participants, 168 (24.6%) were successfully recruited (shown in Fig. 1). A total of 138 participants finally attended the intervention sessions, including 82 women and 56 men, with a mean age of 35.94 (SD = 11.05) years. Of all the recruited participants, 17.9% (14 and 16 in the MBCT-A and CBT-A groups, respectively) did not attend any intervention sessions. There was no difference in this proportion between the two groups. This was viewed as pretreatment attrition and was not included in the data analyses.

The basic participant demographics and baseline clinical data for both groups are listed in Table 1. The baseline characteristics were not significantly different between the MBCT-A group (*n* = 69) and CBT-A group (*n* = 69).

There were 58 (84.1%) and 56 (81.2%) participants in the MBCT-A and CBT-A groups, respectively, who attended at least 5 out of the 8 sessions. The reasons for those dropouts included the following: more than 3 sessions absent without reason, time restriction (i.e., claiming that work or school was too busy to complete the intervention), withdrawn consent without reason after the first session, and suspicious pulmonary tuberculosis after the third session (shown in Fig. 1). Compared with the 114 participants who completed the treatment, the 24 participants who dropped out had a shorter course of GAD (*p* = 0.005) but no significant differences in other characteristics at baseline. The dropout rates in the MBCT-A and CBT-A groups were 15.9% and 18.8% at 8 weeks (*p* = 0.653) and 18.8% and 21.7% at the 3-month follow-up (*p* = 0.672), respectively.

**Table 1:** Baseline participant data

		MBCT-A (n = 69)	CBT-A (*n* = 69)	*P*
**Age, years, mean (SD)**		35.1 (10.1)	36.8 (11.9)	0.373^a^
**Female, n (%)**		38 (55.1)	44 (63.8)	0.386
**Education, years, mean (SD)**		15.7 (3.6)	15.1 (3.2)	0.331^a^
**Marital status, n (%)**	**single**	30 (43.5)	19 (27.5)	0.145
	**married**	36 (52.2)	46 (66.7)	
	**divorced**	3 (4.3)	4 (5.8)	
**Ethnicity(Han), n (%)**		63 (91.3)	68 (98.6)	0.115
**Location (city), n (%)**		66 (95.7)	66 (95.7)	1.000
**Religion (none-religious), n (%)**		57 (82.6)	59 (85.5)	0.817
**Age of onset, years, mean (SD)**		29.5 (10.1)	30.6 (11.5)	0.534^a^
**Course of GAD, months, median (IQR)**		36.0 (71.0)	42.0 (77.0)	0.501^b^
**HAMA, median (IQR)**		22.0 (11.0)	21.0 (10.0)	0.377^b^
**HAMD, mean (SD)**		11.5 (5.1)	11.2 (4.7)	0.653^a^
**CGI-S, median (IQR)**		4.0 (1.0)	4.0 (3.0)	0.682^b^
**STAI-state, median (IQR)**		52.0 (28.0)	49.0 (24.0)	0.295^b^
**STAI-trait, mean (SD)**		55.7 (12.5)	53.7 (10.7)	0.326^a^
**SF-12, mean (SD)**		21.2 (6.3)	23.1 (6.0)	0.071^a^
**FFMQ, mean (SD)**		111.3 (14.8)	112.0 (17.0)	0.782^a^
**Use of antidepressants, n (%)**	**SSRI**	50 (72.5)	53 (76.5)	0.696
	**SNRI**	19 (27.5)	16 (23.2)	
**Use of benzodiazepines, n (%)**		24 (34.8)	24 (34.8)	1.000
**Use of atypical antipsychotics, n (%)**		11 (15.9)	7 (10.1)	0.449

*p < 0.05. a Independent samples t-test. b Mann-Whitney U test. *SD *standard deviation

### Primary outcomes

ITT analysis, including 138 participants who attended at least one session, showed a response rate at 8 weeks of 72.5% for MBCT-A and 65.2% for CBT-A, whereas the PP analysis, including 114 participants who attended at least five out of the eight sessions, showed a response rate at 8 weeks of 86.2% for MBCT-A and 80.4% for CBT-A. The rate differences for MBCT-A compared with CBT-A were 7.25% (95% CI: -8.16, 22.65) in ITT analysis and 5.85% (95% CI: -7.83, 19.53) in PP analysis (shown in Table 2). Both ITT and PP analyses showed that MBCT-A was noninferior compared with CBT-A in terms of the response rate, as the 95% CI of the difference between the two groups was above the predefined noninferiority margin of rate difference (-10%).

**Table 2:** Primary outcomes: HAMA response rate at 8 weeks

Response rate at 8 weeks	MBCT-A n (%)	CBT-A n (%)	Rate difference	95% CI rate difference
**ITT**	50 (72.5)	45 (65.2)	7.25	-8.16,22.65
**PP**	50 (86.2)	45 (80.4)	5.85	-7.83,19.53

*ITT* Intention-to-treat, *PP *per-protocol

### Secondary outcomes

At 8 weeks, the HAMA remission rate in the MBCT-A group was significantly higher than that in the CBT-A group (63.8% in the MBCT-A group vs. 44.6% in the CBT-A group, *p* = 0.040, Cohen’s d = 0.39) (shown in Table 3).

At the 3-month follow-up assessment, neither the remission rate (48.2% in the MBCT-A group vs. 48.1% in the CBT-A group, *p* = 0.994, Cohen’s d = 0.19) nor the response rate (80.4% in the MBCT-A group vs. 74.1% in the CBT-A group, *p* = 0.432, Cohen’s d = 0.00) were significantly different between the two groups (shown in Table 3).

**Table 3:** Secondary outcomes: response rates and remission rates in the two groups using the χ2 test

	MBCT-A	CBT-A	Effect size	*P* value	
**8 weeks**	*N* = 58	*N* = 56	(Cohen’s d)		
**HAMA remission n (%)**	37 (63.8)	25 (44.6)	0.392	0.040*	
**3 months**	*N* = 56	*N* = 54			
**HAMA response n (%)**	45 (80.4)	40(74.1)	0.150	0.432	
**HAMA remission n (%)**	27 (48.2)	26 (48.1)	0.191	0.994	

*p < 0.05

The two-way mixed ANOVAs with time as the repeated measure, intervention group as the between-subjects factor, and HAMA total score, HAMA psychic score, HAMA somatic score, HAMD score, and SF-12 score as the dependent variables all revealed a significant main effect of time but no significant intervention group × time interaction (shown in Table 4). To further explore the time effect, we performed pairwise comparisons with the Bonferroni correction. Both groups showed significant improvements in HAMA total, psychic, and somatic scores, HAMD scores, and SF-12 scores between the baseline and immediate after-treatment assessments (T1 to T2) and between the baseline and 3-month follow-up assessments (T1 to T3) (shown in Online Supplementary Table 1).

The two-way mixed ANOVAs with time as the repeated measure, the intervention group as the between-subjects factor, and STAI-S, STAI-T, CGI-S, and FFMQ scores as dependent variables revealed a significant main effect of time and a significant intervention group × time interaction (shown in Table 4). Therefore, a simple effects analysis was performed in STAI-S, STAI-T, CGI-S, and FFMQ scores (shown in Online Supplementary Table 2).

**Table 4:** Secondary outcomes: the two-way mixed ANOVA results

variable	Main effects for time			Main effects for group		Interaction (Time×Group)		
	**F**	**P**	**partial η2**	**F**	**P**	**F**	**P**	**partial η2**
**HAMA total**	302.98	< 0.001	0.737	0.26	0.610	2.14	0.127	0.019
**HAMA psychic**	198.63	< 0.001	0.648	0.01	0.912	1.78	0.171	0.016
**HAMA somatic**	237.60	< 0.001	0.687	0.56	0.455	1.38	0.255	0.013
**HAMD**	82.79	< 0.001	0.434	0.02	0.888	1.16	0.317	0.011
**CGI-S**	195.80	< 0.001	0.645	2.94	0.089	5.80	0.004*	0.051
**STAI-S**	80.25	< 0.001	0.426	0.11	0.741	3.56	0.030*	0.032
**STAI-T**	108.74	< 0.001	0.502	0.18	0.671	4.28	0.015*	0.038
**SF-12**	99.94	< 0.001	0.481	0.27	0.603	2.03	0.134	0.018
**FFMQ**	53.55	< 0.001	0.331	1.28	0.261	5.26	0.006*	0.046

Listwise deletion resulted in a final sample size of *n* = 110

The CGI-S scores revealed significant group simple effects at the 8-week assessment, F(1,53) = 11.403, *P* = 0.001. This indicates that the MBCT-A group exhibited a significant greater decrease in CGI-S scores compared with the CBT-A group immediately after the intervention. However, the GGI-S scores revealed no significant difference between the MBCT-A and CBT-A groups at the three-month follow-up assessment, F(1,53) = 0.649, *P* = 0.424. Additionally, the CGI-S scores revealed significant time simple effects between the 8-week assessment and the three-month follow-up assessment (*p* = 0.02). Both of these results indicate that the enhanced improvements in MBCT-A had not persisted at the three-month follow-up assessment.

Comparing the FFMQ scores revealed a significant simple effect of group at 8 weeks, F(1,53) = 5.104, *P* = 0.028. Thus, while the level of mindfulness increased in both groups immediately posttreatment, the increase was significantly greater in the MBCT-A group. There were no significant group simple effects in STAI-S and STAI-T scores at the 8-week assessment and no significant group simple effects in any STAI-S, STAI-T, CGI-S, or FFMQ scores at the 3-month follow-up assessments.

Mean values for all outcomes at all time points are reported for patients who completed the entire trial (shown in Online Supplementary Table 3). No severe adverse events were identified.

## Discussion

To the best of our knowledge, this is the first noninferiority RCT study to compare MBCT with high-intensity, evidence-based group CBT, which is considered the first-line psychotherapy for the treatment of GAD [5, 11, 41].

The main finding of the present RCT is that MBCT-A is noninferior to CBT-A, the first-line psychological treatment of GAD, in improving symptoms of anxiety among GAD patients after 8 weeks intervention. In this study, the ITT, as well as PP analyses, both showed that GAD patients who received MBCT-A were noninferior compared with those who received CBT-A after the 8-week treatment period [ITT response rate difference = 7.25% (95% CI: -8.16, 22.65); PP response rate difference = 5.85% (95% CI: − 7.83, 19.53)]. After treatment, 86.2% of the patients in the MBCT-A group and 80.4% of the patients in the CBT-A group achieved a response (shown in Table 2). The result was consistent with a previous study compared MBCT with a low-intensity intervention using CBT principles and TAU. Wong et al [27] conducted an RCT to evaluate the effectiveness of MBCT in reducing anxiety among people with GAD. They reported that both MBCT and psychoeducation, which was assumed to be low-intensity intervention, were better than TAU in reduction of anxiety. They found no statistically significant difference in Beck Anxiety Inventory scores between the psychoeducation and MBCT groups. The results of current study and the previous study indicates that MBCT-A is a promising alternative to CBT-A in treating GAD.

Previous meta-analytic review on effects of MBI reported that the pre-post treatment effects of MBI on anxiety were robust and unlikely to be the results of a psychological placebo [18]. In addition, GAD is a chronic condition that is unlikely to improve naturally over time [42]. As a result, in current study, we designed a two-arm noninferiority study to directly compare the effects of MBCT-A with CBT-A for treating GAD patients and did not include a waitlist control group.

Four additional exploratory findings were obtained from the present study.

First, both MBCT-A group and CBT-A group remained effective at the 3-month follow-up visit. On follow-up, 80.4% of the patients in the MBCT-A group and 74.1% of the patients in the CBT-A group remained a response, and 48.2% and 48.1%, respectively, remained remission. There was no statistically significant in HAMA response rate (*p* = 0.432) and in HAMA remission rate (*p* = 0.994) at the 3-month follow-up visit (shown in Table 3). Given that all the participants had significant anxiety symptoms (HAMA scores higher than 14) under TAU at baseline, we interpreted the improvement as an effect of the treatment.

**Fig. 2:**
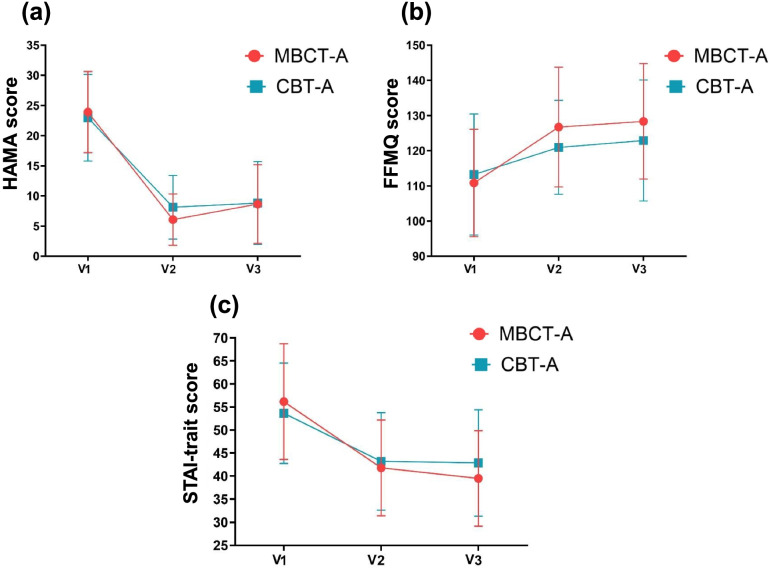
Estimated marginal means for HAMA (**a**), FFMQ (**b**) and STAI-trait (**c**) scores across time for MBCT-A and CBT-A groups. Error bars represent the 95% confidence intervals.

Second, the two groups had a different trend in HAMA remission rate during the treatment and follow-up. First, at the 8-week assessment, the MBCT-A group had a significantly higher remission rate compared with CBT-A group:63.8% in MBCT-A and 44.6% in CBT-A (*p* = 0.040). Second, there was a drop (63.8–48.2%) in the HAMA remission rate in the MBCT-A group but not the CBT-A group (44.6–48.1%) (shown in Table 3; Fig. 2). One plausible explanation for more improvements after treatment is the request that participants in the MBCT-A group report their mindfulness home practice in a WeChat group on a daily basis, which could have promoted engagement. It is widely accepted that for MBI participants, there is a significant association between the extent of practice and positive intervention outcomes [43],especially concerning anxiety [44]. In contrast, in the CBT-A group, weekly homework was handed in to the therapists and discussed in the following session. Thus, this difference in the homework expectations may account for the advantage of MBCT-A at the 8-week visit. While both groups remained effective at the 3-month follow-up, the CBT-A group appeared to show better stability of effectiveness between 8-week and 3-month follow-ups. A previous study [29] found that, compared with psychoeducation with exercise control, MBCT led to short-term but not long-term benefits for patients with chronic insomnia. This is not surprising, as vigorous practice is essential for the beneficial effects of MBCT. These data indicate that MBCT-A should not be delivered in a one-time or short-term way, but rather, a long-term pattern should be encouraged and integrated via deliberate lifestyle modification.

Third, unlike anxiety symptoms, the trait-related outcomes, including trait anxiety and trait mindfulness, showed further improvements at the 3-month follow-up visit compared with at 8 weeks, although this was not statistically significant (shown in Fig. 2). A previous study found similar changes in anxiety symptoms among GAD patients randomly assigned to MBCT, CBT-based psychoeducation, or usual care [27, 29]. Thus, the benefits of MBCT may not be limited to symptomology but may promote a positive change in personality. Indeed, we found a significant improvement in the quality of life, which was reflected in the change in SF-12 scores. To some extent, MBCT may reflect cultural context, core values, and attitudes regarding life.

Fourth, MBCT-A was effective in improving a wider range of outcomes, including well-being, overall illness severity, state and trait anxiety symptoms and depression symptoms. These findings are not only concordant with our hypothesis, but are also in line with previous studies demonstrating the effectiveness of MBCT in treating GAD [27, 29].

Our study has several strengths. To our knowledge, this is the first noninferiority RCT study to compare MBCT and high-intensity CBT among Chinese GAD patients. Additionally, there was high homogeneity in the participant diagnoses, adding to the generalizability of the findings. Furthermore, we adapted MBCT for treating GAD and the interventions were delivered by trained and experienced instructors in accordance with standard protocols, thus demonstrating implementation of the treatment for future reference.

Our study also has several limitations. First, we did not have a no-treatment control or a wait-list control. Current evidence suggests that GAD is a chronic condition that is unlikely to improve naturally over time [1]. Considering this and considering that we were comparing a new treatment with a well-established one, we thought it more ethical to provide treatment to both groups [45]. Second, due to practical difficulties, we did not record the specific reasons for screen failure during the recruitment process. In addition, we did not collect data on the mindfulness exercises in the MBCT-A group or the behaviour exercises in the CBT-A group. Third, as the two groups had multiple and overlapping specific and nonspecific components, which components led to the positive effects was not clear. Further study is necessary to elucidate this point. Finally, we did not match the total treatment time between the two groups. As shown in a previous study [46], more treatment time may produce larger effects. We chose not to match the treatment time between the two groups in consideration of clinical practical applications.

## Conclusions

MBCT-A was noninferior to CBT-A in reducing anxiety symptoms in GAD patients. Both interventions appeared to be effective for long-term benefits. Taking into account the differences in treatment priorities and components between MBCT-A and CBT-A, our results suggest that both evidence-based interventions are optional treatments for patients with GAD.

## Supplementary Information

**Additional file 1.**

**Additional file 2.**

**Additional file 3.**

## Data Availability

All data needed to support the conclusions in the paper are presented in the paper and/or the Supplementary Materials.

## Abbreviations

MBCT: mindfulness-based cognitive therapy
GAD: generalized anxiety disorder
MBCT-A: MBCT adapted for treating GAD
CBT: cognitive behavioural therapy
CBT-A: CBT designed to treat GAD
TAU: treatment-as-usual
HAMA: Hamilton Anxiety Scale
STAI: the State-Trait Anxiety Inventory
HAMD: Hamilton Depression Scale
CGI-S: Clinical Global Impression Scale
SF-12: 12-item Short-Form Health Survey
FFMQ: Five Facet Mindfulness Questionnaire
DSM-IV: Diagnostic and Statistical Manual of Mental Disorders, fourth edition
LB: lower bound
UB: upper bound
SD: standard deviation
SE: standard error
ITT: intention-to-treat
PP: per-protocol
