# Supplementary material for: Effects of group mindfulness-based cognitive therapy and group cognitive behavioural therapy on symptomatic generalized anxiety disorder: a randomized controlled noninferiority trial
Source: BMC Psychiatry. 2022 Jul 19;22:481. doi: 10.1186/s12888-022-04127-3 (PMC9295460; doi:10.1186/s12888-022-04127-3)
Supplement: Supplementary file 1 — Additional file 1. [file 12888_2022_4127_MOESM1_ESM.doc]

| **variable** | **Comparisons** | | **Mean difference**  **(A-B)** | | **SE P** | | **95% CI of difference**  **LB UB** | |
| --- | --- | --- | --- | --- | --- | --- | --- | --- |
|  | **Time A** | **Time B** | |  |  |  |  |  |
| **HAMA total** | baseline | 8weeks | | 16.346 | 0.719 | 0.000 | 14.597 | 18.094 |
|  |  | 3month | | 14.708 | 0.849 | 0.000 | 12.644 | 16.772 |
|  | 8weeks | 3month | | -1.637 | 0.606 | 0.024* | -3.111 | -0.164 |
| **HAMA psychic** | baseline | 8weeks | | 8.466 | 0.446 | 0.000 | 7.382 | 9.551 |
|  |  | 3month | | 7.705 | 0.561 | 0.000 | 6.342 | 9.068 |
|  | 8weeks | 3month | | -0.761 | 0.387 | 0.155 | -1.702 | 0.179 |
| **HAMA somatic** | baseline | 8weeks | | 7.861 | 0.423 | 0.000 | 6.381 | 8.890 |
|  |  | 3month | | 7.031 | 0.443 | 0.000 | 5.952 | 8.109 |
|  | 8weeks | 3month | | -0.830 | 0.308 | 0.025* | -1.580 | -0.080 |
| **HAMD** | baseline | 8weeks | | 6.821 | 0.539 | 0.000 | 5.510 | 8.132 |
|  |  | 3month | | 5.458 | 0.643 | 0.000 | 3.895 | 7.022 |
|  | 8weeks | 3month | | -1.363 | 0.490 | 0.019* | -2.555 | -0.170 |
| **SF-12** | baseline | 8weeks | | -6.079 | 0.590 | 0.000 | -7.513 | -4.645 |
|  |  | 3month | | -7.724 | 0.638 | 0.000 | -9.275 | -6.172 |
|  | 8weeks | 3month | | -1.645 | 0.489 | 0.003* | -2.834 | -0.455 |

**Online Supplementary Table 1 Pairwise comparison between time points for HAMA total, HAMA psychic, HAMA somatic, HAMD, and SF-12 scores**.

The Bonferroni adjustment was used for multiple comparisons. * means p ＜0.05

Abbreviations: A-B: Time A compared to Time B; LB: lower bound; UB: upper bound; SE: standard error; HAMA, Hamilton Anxiety Scale; Hamilton Depression Rating Scale, HAMD; 12-item Short-Form Health Survey, SF-12
