# Supplementary material for: Effects of group mindfulness-based cognitive therapy and group cognitive behavioural therapy on symptomatic generalized anxiety disorder: a randomized controlled noninferiority trial
Source: BMC Psychiatry. 2022 Jul 19;22:481. doi: 10.1186/s12888-022-04127-3 (PMC9295460; doi:10.1186/s12888-022-04127-3)
Supplement: Supplementary file 2 — Additional file 2. [file 12888_2022_4127_MOESM2_ESM.doc]

**Online Supplementary Table 2. Pairwise comparisons between time points for each treatment group in terms of CGI-S, STAI-S, STAI-T, and FFMQ scores**

| **Group** | **variable** | **Comparisons** |  | | **Mean difference**  **(A-B)** | | **SE P** | | **95% CI of difference**  **LB UB** | |
| --- | --- | --- | --- | --- | --- | --- | --- | --- | --- | --- |
| **MBCT-A** |  | **Time A** | | **Time B** | |  |  |  |  |  |
|  | **CGI-S** | baseline | | 8weeks | | 2.107 | 0.176 | 0.000 | 1.673 | 2.542 |
|  |  |  | | 3month | | 1.696 | 0.127 | 0.000 | 1.382 | 2.011 |
|  |  | 8weeks | | 3month | | -0.411 | 0.146 | 0.020* | -0.771 | -0.050 |
|  | **STAI-S** | baseline | | 8weeks | | 16.286 | 2.085 | 0.000 | 11.137 | 21.435 |
|  |  |  | | 3month | | 15.268 | 2.178 | 0.000 | 9.889 | 20.647 |
|  |  | 8weeks | | 3month | | -1.018 | 1.180 | 1.000 | -3.931 | 1.895 |
|  | **STAI-T** | baseline | | 8weeks | | 14.375 | 1.892 | 0.000 | 9.703 | 19.047 |
|  |  |  | | 3month | | 16.661 | 1.919 | 0.000 | 11.923 | 21.398 |
|  |  | 8weeks | | 3month | | 2.286 | 1.015 | 0.085 | -0.220 | 4.792 |
|  | **FFMQ** | baseline | | 8weeks | | -15.893 | 2.457 | 0.000 | -21.961 | -9.825 |
|  |  |  | | 3month | | -17.518 | 2.223 | 0.000 | -23.007 | -12.028 |
|  |  | 8weeks | | 3month | | -1.625 | 1.793 | 1.000 | -6.053 | 2.803 |
| **CBT-A** |  | **Time A** | | **Time B** | |  |  |  |  |  |
|  | **CGI-S** | baseline | | 8weeks | | 1.444 | 0.131 | 0.000 | 1.120 | 1.769 |
|  |  |  | | 3month | | 1.444 | 0.134 | 0.000 | 1.113 | 1.776 |
|  |  | 8weeks | | 3month | | 0.000 | 0.106 | 1.000 | -0.261 | 0.261 |
|  | **STAI-S** | baseline | | 8weeks | | 9.944 | 1.552 | 0.000 | 6.108 | 13.781 |
|  |  |  | | 3month | | 11.259 | 1.771 | 0.000 | 6.881 | 15.637 |
|  |  | 8weeks | | 3month | | 1.315 | 1.080 | 0.686 | -1.354 | 3.984 |
|  | **STAI-T** | baseline | | 8weeks | | 10.444 | 1.192 | 0.000 | 7.497 | 13.392 |
|  |  |  | | 3month | | 10.759 | 1.370 | 0.000 | 7.271 | 14.147 |
|  |  | 8weeks | | 3month | | 0.315 | 0.945 | 1.000 | -2.022 | 2.652 |
|  | **FFMQ** | baseline | | 8weeks | | -7.722 | 2.018 | 0.001 | -12.711 | -2.734 |
|  |  |  | | 3month | | -9.667 | 2.018 | 0.000 | -14.656 | -4.677 |
|  |  | 8weeks | | 3month | | -1.944 | 1.394 | 0.507 | -5.391 | 1.502 |

The Bonferroni adjustment was used for multiple comparisons. * means p ＜0.05

Abbreviations: MBCT-A, mindfulness cognitive therapy adapted for treating GAD; CBT-A, cognitive behavioural therapy designed to treat GAD; CGI-S, Clinical Global Impression-Severity; STAI-S, State Anxiety Inventory; STAI-T, Trait Anxiety Inventory ; FFMQ, Five Facet Mindfulness Questionnaire
