# Supplementary material for: Effects of group mindfulness-based cognitive therapy and group cognitive behavioural therapy on symptomatic generalized anxiety disorder: a randomized controlled noninferiority trial
Source: BMC Psychiatry. 2022 Jul 19;22:481. doi: 10.1186/s12888-022-04127-3 (PMC9295460; doi:10.1186/s12888-022-04127-3)
Supplement: Supplementary file 3 — Additional file 3. [file 12888_2022_4127_MOESM3_ESM.doc]

**Online Supplementary Table 3 Mean values and SD’s for each group for all outcomes at all time points**

| **variable** | **group** | **Baseline,**  **Mean (SD)**  **N=138**^a^ | **8-weeks,**  **Mean (SD)**  **N=114**^b^ | **3month,**  **Mean (SD)**  **N=110**^c^ |
| --- | --- | --- | --- | --- |
| **HAMA total** | MBCT-A | 24.09(7.085) | 6.69(5.109) | 8.66（6.548） |
|  | CBT-A | 23.28(7.096) | 8.02(5.255) | 8.81（6.907） |
| **HAMA psychic** | MBCT-A | 12.97（4.243） | 4.03（3.843） | 5.04（4.112） |
|  | CBT-A | 12.81（3.960） | 4.61（3.441） | 4.81（3.660） |
| **HAMA somatic** | MBCT-A | 11.13（4.112） | 2.67（2.598） | 3.62（3.012） |
|  | CBT-A | 10.46（4.708） | 3.45（2.558） | 3.96（3.741） |
| **HAMD** | MBCT-A | 11.54(5.109) | 4.05(3.873) | 5.77（5.034） |
|  | CBT-A | 11.16(4.708) | 4.66(3.810) | 5.52（4.769） |
| **STAI-S** | MBCT-A | 53.12（14.895） | 37.45（12.056） | 38.18（10.647） |
|  | CBT-A | 50.59（14.236） | 41.00（11.941） | 39.39（12.460） |
| **STAI-T** | MBCT-A | 55.65（12.514） | 42.10（11.051） | 39.50（10.368） |
|  | CBT-A | 53.70（10.749） | 43.30（10.472） | 42.87（11.542） |
| **SF-12** | MBCT-A | 21.17（6.271） | 28.43（5.774） | 29.77(5.812 |
|  | CBT-A | 23.07（5.974） | 27.89（6.172） | 30.09(6.104) |
| **CGI-S** | MBCT-A | 4.39(0.771) | 2.38（1.152） | 2.75(0.919 |
|  | CBT-A | 4.32(0.776) | 2.91（0.721） | 2.91(0.853) |
| **FFMQ** | MBCT-A | 111.25（14.815） | 126.64（17.559） | 128.36(16.397) |
|  | CBT-A | 112.00（17.000） | 120.86（13.840） | 122.91(17.186) |

Abbreviations: SD: standard deviation; MBCT-A: mindfulness cognitive therapy adapted for treating GAD; CBT-A: cognitive behavioural therapy designed to treat GAD; HAMA: Hamilton Anxiety Scale; HAMD: Hamilton Depression Scale; STAI: the State-Trait Anxiety Inventory; SF-12: 12-item Short-Form Health Survey; CGI-S: Clinical Global Impression Scale; FFMQ: Five Facet Mindfulness Questionnaire

a all participants who finally attended the intervention sessions

b participants who completed the 8-week treatment

c participants who completed the assessment 3 months follow-ups
